# Behavioral measures to fight COVID-19: An 8-country study of perceived usefulness, adherence and their predictors

**DOI:** 10.1371/journal.pone.0243523

**Published:** 2020-12-07

**Authors:** Jürgen Margraf, Julia Brailovskaia, Silvia Schneider

**Affiliations:** 1 Mental Health Research and Treatment Center, Department of Clinical Psychology and Psychotherapy, Ruhr-Universität Bochum, Bochum, Germany; 2 Mental Health Research and Treatment Center, Department of Clinical Child and Adolescent Psychology, Ruhr-Universität Bochum, Bochum, Germany; University of Birmingham, UNITED KINGDOM

## Abstract

Behavioral measures, such as the wearing of facemasks and maintaining of distance to other people, have been central in fighting the COVID-19 pandemic and will continue to be important in curbing its spread. We therefore investigated their perceived usefulness, adherence and their predictors in representative online samples in eight countries (France, Germany, Poland, Russia, Spain, Sweden, U.K., U.S.). Of the 7,658 participants, 77.4% rated governmental measures (highest: Germany, lowest: France) as useful and 91.7% reported adherence to them. Adherence was lowest in Russia and Poland, where people felt particularly left alone and not well supported, and in the U.S. and Sweden, where governments showed ambivalent attitudes towards the measures. The highest adherence was reported in countries with very high mortality (U.K., Spain, France) or very positively perceived government communication (Germany). Female gender, higher age, belonging to a risk group, being affected physically and mentally, perception of governmental communication as guided by the interests of people, feeling of being well informed and the level of positive mental health positively predicted both outcomes, while being affected economically negatively predicted both outcomes. Country-specific results are considered in the light of the protection motivation theory and the theory of planned behavior together with potential ways to improve active participation of the population. Overall, we recommend the governments and authorities to stress that each individual can contribute to the control of the COVID-19 situation by adherence to the measures in the public communication. Moreover, they should emphasize the risk of unconscious infection of older individuals by younger people, as well as the importance of physical activity for the protection of mental and physical health especially during the pandemic.

## Introduction

In March 2020, the coronavirus disease 2019 (COVID-19; severe acute respiratory syndrome coronavirus 2, SARS-CoV-2; a list of used abbreviations is available in S1 Abbreviations) was recognized as a pandemic [1]. By November 17, 2020, COVID-19 had spread across 217 countries or territories, with 55.18 million confirmed infections and 1.33 million deaths [2]. The rapid spread and significant mortality rate of COVID-19, particularly among older people, prompted governments to take drastic measures. In view of the lack of specific drug therapies or vaccinations, behavioral measures, such as the wearing of facemasks and maintaining of distance to other people, were of crucial importance.

Beginning in March of 2020, national lockdowns were declared by many governments [3]. The exact extent and timing of virus spread reduction measures varied between and within countries. However, most countries included limitations on non-family gatherings in public places (e.g., in France for more than ten people; in some federal states of Germany and in Poland for more than two people), bans on travel, cancelation of mass events, requests for home-office and home-schooling; temporary closure of public establishments, schools and universities, non-essential businesses, entertainment and recreation venues [4–6]. Some governments and authorities issued a “stay-at-home” order for all residents (e.g., some states/provinces in the U.S., in Russia, in France, in Spain), except to purchase food and medicine, essential work, or short walks with pets [7–10]. Others encouraged the population to “stay-at-home” but did not order it (e.g., some states in Germany; at the beginning of the pandemic in the U.K.) [11,12]. All of the measures aimed to slow down the spread of the virus by reducing physical contact within the population, so-called “social distancing” [13]. Since May of 2020, many countries began to ease or lift some measures of the lockdown by re-opening public establishments and recreation areas, again allowing larger public gatherings. Nevertheless, other measures such as the wearing of facemasks (on public transport, in shops and/or in all public places) and the keeping of distance from other people have been maintained or reintroduced in the light of renewed increases in infection rates [10,14,15].

The success of these government measures depends heavily on the willingness of the population to actively participate [16]. Here, however, there may be considerable differences within and across countries: While some individuals consider the measures to be useful and adhere to the guidelines, others question them and do not comply [17,18]. Moreover, the extent of COVID-19 exposure, government countermeasures and their communication vary considerably across countries. Therefore, the comparison of different countries promises a significant additional gain in knowledge. Against this background, there is an urgent need to examine the attitudes and behaviors leading to (non-)adherence together with their predictors in an international comparison. This can not only help to identify persons at increased risk of non-adherence, but also to find starting points for improving the cooperation of the population in the sustainable control of COVID-19. For this reason, the present study examined the extent to which the measures to combat COVID-19 are considered useful by the population and to what extent they are being adhered to across different countries. In addition, potential predictors of these two variables were investigated.

Previous research has shown that the protection motivation theory (PMT; [19]) provides a useful framework on intention formation and behavioral choices during pandemics and extraordinary societal situations [20–23]. According to PMT, the self-protection motivation in the case of a threat depends on (1) threat appraisal (that is perceived severity of the threat; perceived personal vulnerability/risk; emotional response to the threat) and (2) coping appraisal (that is perceived response-efficacy–own belief about the effectiveness of the recommended protective behavior; perceived self-efficacy–own belief about the ability to adopt the recommended behavior; perceived costs of the recommended behavior) [22,24]. High levels of threat appraisal (specifically severity and vulnerability) and of efficacy (response- and self-), and low levels of perceived costs were figured out to be the most important predictors of the perception of usefulness and the adaptation to the recommended behavior [22,25].

On the basis of PMT, the following variables can be considered as expressions of threat or coping appraisal and thus act as potential predictors of evaluation of anti-COVID-19 measures and adherence to them. Threat Appraisal: Low-perceived physical health and belonging to a high-risk group for COVID-19 (e.g., higher age, pre-existing conditions, weakened immune system) are expected to enhance the perception of threat severity and own vulnerability [24]. Individuals who are predisposed to specific health problems typically show better adherence to preventive measures [26]. In addition, the level of experienced physical and mental burden linked to the COVID-19 pandemic represents the emotional response to threat appraisal. Social distancing and public restrictions significantly changed the pattern of everyday life in ways that can negatively influence self-reported physical and mental health [16]. Increased depression and anxiety symptoms, problematic substance use as well as child abuse and domestic violence have been predicted in recent literature [27]. Thus, low levels of perceived physical health, being in a COVID-19 high-risk group, and reporting higher physical and mental burden represent higher threat appraisal and should therefore predict a higher usefulness evaluation of the measures and enhanced adherence.

Coping appraisal is the second relevant PMT construct: The perception of (response- and self-) efficacy are enhanced considerably by positive mental health (PMH) which has been defined as social, emotional and psychological well-being [28]. In a series of studies, PMH confers resilience. It buffers the negative effect of daily stress [29], reduces adjustment disorder symptoms after the experience of stressful life events [30], positively predicts remission from panic disorder, agoraphobia and specific phobia in outpatients [31], reduces the negative impact of problematic media use [32], and decreases suicide related-outcomes [33,34]. Most recently, PMH was shown to predict a more adaptive response to the COVID-19 situation in a prospective longitudinal study [16]. Thus, it can be hypothesized that higher levels of PMH reflect a higher coping appraisal and therefore predict higher usefulness evaluation of the measures and better adherence.

Furthermore, the available literature on previous disease threats emphasizes that the form of public risk communication can significantly impact the population trust in the governments and authorities and the response to the anti-COVID-19 measures [35]. A recent cross-national study on COVID-19 confirmed these findings. Across different countries individuals who trusted their governments were more likely to agree that they will accept a vaccine when it is available and that they will respond positively to vaccine recommendation of their employer than people who reported a low level of trust [36]. Effective communication by national governments and authorities, which enhances public confidence and adherence, provides transparent, clear and credible information on the seriousness of the threat, the purpose and the effectiveness of the required actions. The benefits of the actions and the importance of adherence of each individual and the overall population are stressed and it is emphasized that the actions are guided by population interests [20,37,38]. In a recently published cross-national study the belief that taking health precautions will be effective for avoiding COVID-19 positively predicted voluntary health compliance behavior [18]. Therefore, in the current COVID-19 situation, it is of specific importance for public communication to include the explanation why and how health precautions are effective to fight the pandemic. This form of governmental communication fosters the feeling of being taken seriously. This positive experience increases solidarity, commitment and perception of common identity among the population that contribute to the development of a collective resilience to combat the threat [39,40]. Moreover, it can foster response- and self-efficacy and reduce the perceived costs of the needed behaviors [20]. And it can increase the positive evaluation of the governmental reactions during the pandemic outbreak. In a further recently published study that investigated data from Brazil, Colombia, Germany, Israel, Norway, and the U.S., the individual satisfaction with the governmental reactions during the pandemic significantly predicted the perception of efficacy of the introduced restrictions [41]. Thus, it is assumed that the perception of constructive public communication by government and authorities predicts a higher usefulness evaluation of the measures and greater adherence. In the longer term, it can contribute to a higher acceptance of anti-COVID-19 vaccine [36]. In contrast, many individuals are economically affected by the lockdown [42], so that its higher perceived cost may lead them to disagree with the measures. Individuals who are particularly affected by the negative economic effects of the lockdown might therefore be more likely to question the measures and show less adherence.

Based on these considerations and findings, two exploratory questions and two hypotheses were formulated in the current study:

Research Question 1: To what extent are the measures of the governments and authorities to combat the spread of COVID-19 evaluated as useful by the population?

Research Question 2: To what extent are the rules and/or recommendations established by the governments and authorities to combat the spread of COVID-19 adhered to by the population?

Considering the predictors of population response to the measures, it is expected that the usefulness evaluation of the measures and the level of adherence are both positively predicted by belonging to the COVID-19 risk group, lower perceived physical health, a higher level of experienced physical and mental consequences of the COVID-19 outbreak, a constructive form of national public communication regarding the COVID-19 situation (i.e., clear, understandable, credible, honest, guided by the interests of people, feeling of being well supported, well informed and taken seriously by government and authorities), and a higher level of PMH (Hypothesis 1). Finally, being more negatively affected by the economic consequences of the lockdown and the feeling of being left alone by government and authorities are assumed to negatively predict the usefulness evaluation and the level of adherence (Hypothesis 2).

## Methods

### Procedure and participants

Using the framework of our previous comparisons of countries with different welfare systems (see [43,44] for detailed explanation), we examined participants from the following eight countries: France (FR), Germany (GE), Poland (PL), Russia (RU), Spain (ES), Sweden (SV), the U.K. (UK), and the U.S. (US).

At the time of data collection and at the present time, the eight countries had/have quite different case numbers of COVID-19. Table 1 presents the COVID-19 statistics (case numbers per million of a nation’s population) and governmental measures of the eight countries of June 01 (when data of the present study were collected) and more recent statistics of November 17, 2020.

**Table 1:** COVID-19 statistics in the eight investigated countries (status: June 01, 2020; and November 17, 2020, in parentheses).

	FR	GE	PL	RU	ES	SV	UK	US
**Confirmed cases**	2,325 (30,506)	2,170 (9,736)	628 (19,388)	2,781 (13,506)	5,125 (32,297)	3,801 (18,839)	3,764 (20,486)	5,408 (33,853)
**Deaths**	441 (690)	102 (153)	28 (277)	32 (233)	580 (892)	458 (615)	552 (768)	315 (747)
**First cases**	January	January	March	January	January	January	January	January
**Begin of lockdown**	March	March	March	March	March	-	March	March
**First easing of lockdown**	May	April/May	April	May	April/May	-	May	April
**“Stay-at-home” order (whole country or single states/provinces)**	X	X	X	X	X	-	X	X
**Compulsory wearing of facemasks**	X	X	X	X	X	-	X	X
**Social distancing (1.5 to 2 meters/4.9 to 6.6 feet)**	X	X	X	X	X	-	X	X

France = FR, Germany = GE, Poland = PL, Russia = RU, Spain = ES, Sweden = SV, the U.K. = UK, the U.S. = US; X = Yes; figures present case numbers per million of a nation’s population; figures are based on information provided on [2]; references on further country-specific information are provided in the text; “-” = Sweden: No explicit governmental measures.

The overall sample was comprised of 7,658 participants from eight countries: FR: N = 940, GE: N = 917, PL: N = 924, RU: N = 986, ES: N = 960, SV: N = 922, UK: N = 1,105 and US: N = 904. Demographics of all samples are presented in Table 2. Data were collected within ten days from the end of May to the beginning of June 2020 by the independent social marketing and research institute YouGov via population-based online-panel surveys in the national language of the countries. Participants were recruited from residential populations aged 18 years and above. Age, gender and region stratification were implemented to achieve representativeness. In all countries, participation was compensated by panel-specific tokens that can be converted in voucher or monetary payment. The responsible Ethics Committee approved the implementation of the present study. All required permits and approvals for the data collection in the eight countries were obtained. The study was pre-registered with AsPredicted.org on May 25, 2020 (https://aspredicted.org/e7a9g.pdf). All participants were properly instructed and gave online their informed consent to participate. Power analyses (G*Power program, version 3.1) revealed that the sample sizes are sufficient for valid results (power >.80, α = .05, effect size: f^2^ = .15; cf., [45]). All national regulations and laws regarding human subjects research were followed. The dataset used in the present study is available in S2 Dataset.

**Table 2:** Demographic variables (total and individual samples).

	All	FR	GE	PL	RU	ES	SV	UK	US
**Gender (female, %)**	53	57.7	51	54.7	54.7	51	51	52.2	51.8
**Age groups (%)**									
**18 to 24 years**	8.1	8.5	6.7	9.6	7.8	6.3	6.8	10	8.7
**25 to 34 years**	16.6	14.8	12.8	17.5	20.9	14	20.9	17.9	13.5
**35 to 44 years**	16.5	14.9	14.4	18.9	20.3	21.3	8.8	16.9	15.6
**45 to 54 years**	18.4	18.5	19.8	15.4	17.3	20.9	19	18	18.6
**55 years and older**	40.4	43.3	46.3	38.5	33.7	37.6	44.5	37.1	43.6
**Marital Status (%)**									
**Single**	23.4	21.8	23.8	20.5	16.5	23.3	32.9	26.3	22
**Romantic relationship, not married**	16.4	22.8	14.9	17.3	11.6	18.5	22.5	16	7.6
**Married**	47.8	43.6	45.8	50.4	57.4	48.4	35.8	45.7	55.5
**Widowed, divorced**	12.4	11.8	15.5	11.8	14.5	9.7	8.9	11.9	14.8
**Children (yes, %)**	63.6	67.4	57.1	69.3	74	62.5	61.7	55.6	61.6
**Social Status (%)**									
**Lower class**	5.1	7.6	7.6	3.8	2.8	4.1	5.1	2.7	7.6
**Working class**	22.2	19.3	18.4	15.8	19.1	31.4	20.7	33.8	16.4
**Lower middle class**	25.9	26.7	25.3	32.6	37.3	21	13.6	30.2	19.1
**Middle middle class**	36.8	32.8	38.9	36.3	36.8	36.9	46.6	28.4	39.6
**Upper middle class**	9	12	9.2	8.9	3.4	6.6	12.8	4.9	15.9
**Upper class**	1	1.7	0.5	2.7	0.5	0.1	1.2	-	1.3
**Living Environment (%)**									
**Large city**	42.3	28.9	35.1	48.8	77.3	37.9	47.7	25.5	38.4
**Small city**	35	39.7	36	36.6	19.6	41.7	33	35.4	39.2
**Rural community**	22.7	31.4	28.9	14.6	3.1	20.4	19.3	39.1	22.5

All: N = 7,658; France (FR): N = 940, Germany (GE): N = 917, Poland (PL): N = 924, Russia (RU): N = 986, Spain (ES): N = 960, Sweden (SV): N = 922, the U.K. (UK): N = 1,105, the U.S. (US): N = 904; due to rounding, the sum of the frequencies is not always 100%.

### Measures

#### Demographics

Participants were asked to indicate their gender, age range, marital status, social class, living environment, and whether they had children. The operationalization of the demographic variables bases on recommendations provided in the available literature (e.g., [46–49]).

#### COVID-19 specific content

Participants were asked to rate 1) to what extent they think the measures to combat the COVID-19 crisis are useful, and 2) how much they adhere to the rules introduced to combat it, respectively, on a 5-point Likert scale (0 = not at all, 4 = very strong). Furthermore, they were asked to rate 3) whether they belong to the COVID-19 risk group (i.e., age-related, pre-existing condition, weakened immune system) (0 = no, 1 = yes); 4), to what extent they are affected by the current COVID-19 situation 4a) in terms of physical health, 4b) economically, and 4c) mentally, respectively, on 5-point Likert scales (0 = not at all, 4 = very strong); 5) and to what extent they assess the communication of the national government and authorities regarding the COVID-19 crisis as 5a) clear and understandable, 5b) credible and honest, 5c) guided by the interests of the people, respectively, on 5-point Likert scales (0 = not at all true, 4 = very true). They were also asked to rate to what extent 6) they feel themselves by the national government and authorities 6a) well supported, 6b) well informed, 6c) taken seriously and 6d) left alone, respectively, on 5-point Likert scales (0 = not at all true, 4 = very true).

#### Physical health state

The EuroQuol Visual Analogue Scale (EQ VAS; [50,51])–a visual analogue scale ranging from 0 (worst imaginable physical health state) to 100 (best imaginable physical health state)–measured overall current physical health state.

#### Positive mental health (PMH)

The unidimensional Positive Mental Health Scale (PMH-Scale; [28]) measured the level of PMH. The PMH-Scale is an internationally well-established instrument for the assessment of psychological, emotional and social well-being. Previous research described its strong measurement invariance and encouraged the use of this instrument in cross-cultural studies [52]. The nine items of the PMH-Scale are rated on a 4-point Likert scale (e.g., “I enjoy my life”; 0 = do not agree, 3 = agree). Current scale reliability is Cronbach’s α = .87 (FR) to .93 (PL, SV, UK). Higher sum scores indicate higher levels of PMH.

### Statistical analyses

Statistical analyses were conducted using SPSS 24. The means of the investigated variables were compared between the eight countries by calculating multiple analyses of variance (MANOVAs). Following the recommendation of Stevens [53] not to use more than ten dependent variables in one MANOVA, three analyses were calculated. The variables gender, age group, marital status, children, social status and living environment served as control variables in all analyses. In the first MANOVA, the evaluation of usefulness of measures to combat COVID-19 and the adherence to the rules were included as dependent variables. In the second MANOVA, belonging to the risk group, being affected by the current COVID-19 situation in terms of health, economically and mentally, current physical health state and PMH were included. The third MANOVA included the perception of the COVID-19 communication of government and authorities as clear and understandable, credible and honest, guided by interests of people, the feeling of being well supported, well informed, taken seriously and left alone as dependent variables. The Box’s test was significant in all three analyses, and thus the Hotelling’s trace statistic was used. Partial eta squared (η^2^_p_) was used as the effect-size measure of the main effects, Cohen’s d was used as effect-size measure of the post-hoc comparisons between the countries. Post-hoc comparisons were all Bonferroni-corrected or Tamhane-corrected depending on variance homogeneity (level of significance: p < .05, two-tailed).

The relationship between the evaluation of usefulness of the measures and adherence to them was assessed by a zero-order bivariate correlation analysis. In the next step, two two-step hierarchical regression analyses assessed the predictors of the evaluation of measures introduced to reduce the spread of COVID-19 as useful (regression 1) and of the adherence to rules or recommendations (regression 2). In both models, Step 1 included gender (coded: 0 = woman, 1 = man), age group, marital status, children, social status and living environment as control variables; Step 2 included belonging to the risk group, being affected by the current COVID-19 situation in terms of physical health, economically and mentally, perception of the communication of government and authorities as clear and understandable, credible and honest, guided by interests of people, the feeling of being well supported, well informed, taken seriously and left alone, physical health state and PMH. There was no violation of the multi-collinearity assumption (all values of tolerance >.25, all variance inflation factor values <5; [54]). All calculations were first conducted with the overall sample (N = 7,568) and subsequently with the eight country-specific samples, respectively, in order to identify possible country-specific differences.

## Results

Table 3 (outcomes) and Table 4 (potential predictors) present the descriptive statistics of the investigated variables, in the total sample and for each participating country.

**Table 3:** Descriptive statistics and correlation of the outcomes (total and individual samples).

	All	FR	GE	PL	RU	ES	SV	UK	US
**Perceived usefulness**									
**M (SD)**	2.31(1.16)	2.08(1.08)	2.60(1.16)	2.09(1.13)	2.16(1.21)	2.48(1.14)	2.16(1.20)	2.51(1.00)	2.36(1.24)
**Frequency of ratings (%)**									
**“0” = Not at all**	8.8	10.9	7.6	9.6	11.8	5.6	12.4	3.7	9.5
**“1” = Little**	13.8	14.4	8.1	18.5	15.1	14.8	15.2	10	14.9
**“2” = Moderate**	31.8	38	25.5	36.6	34.8	26.6	30.3	33.9	27.8
**“3” = Strong**	29.2	29.1	34.2	23.6	22.2	32.3	28.9	36.3	26
**“4” = Very strong**	16.5	7.7	24.5	11.7	16.1	20.7	13.3	16	21.8
**Adherence to measures**									
**M (SD)**	2.97 (1.00)	3.07(.94)	3.02(.92)	2.79(1.07)	2.48(1.03)	3.29(.83)	2.85(.94)	3.35(.80)	2.88(1.14)
**Frequency of ratings (%)**									
**“0” = Not at all**	2.8	2.7	2.4	4.4	4	1	2	0.9	5.1
**“1” = Little**	5.5	3.7	3.8	8.1	10.8	2.5	5.6	2.2	7.7
**“2” = Moderate**	18.2	13.4	15.6	19.4	36.6	10.7	23.9	9	18
**“3” = Strong**	39.1	44.5	46.2	40.4	31.1	38.3	42.5	37.4	32.9
**“4” = Very strong**	34.5	35.7	32	27.7	17.5	47.4	26	50.6	36.3
**Correlation (r)**									
**Measures usefulness x Adherence to rules**	.490**	.392**	.630**	.516**	.623**	.425**	.235**	.366**	.636**

All: N = 7,658; France (FR): N = 940, Germany (GE): N = 917, Poland (PL): N = 924, Russia (RU): N = 986, Spain (ES): N = 960, Sweden (SV): N = 922, the U.K. (UK): N = 1,105, the U.S. (US): N = 904; M = Mean; SD = Standard Deviation; due to rounding, the sum of the frequencies is not always 100%;

**p < .001.

**Table 4:** Descriptive statistics of potential predictors (total and individual samples).

	All	FR	GE	PL	RU	ES	SV	UK	US
**Risk group (yes, %)**	38.1	30	46.1	43.2	41.7	31.8	37.3	29.7	47.2
**M (SD)**									
**Affected: Health**	1.02(1.16)	1.11(1.14)	.61(.94)	1.27(1.13)	1.51(1.25)	1.20(1.19)	.98(1.15)	.62(.96)	.93(1.17)
**Affected: Economically**	1.49(1.29)	1.42(1.21)	1.01(1.13)	1.94(1.14)	2.36(1.23)	1.56(1.25)	.99(1.18)	1.15(1.22)	1.46(1.29)
**Affected: Mentally**	1.52(1.17)	1.34(1.12)	1.23(1.07)	1.75(1.17)	1.57(1.26)	1.62(1.10)	1.55(1.15)	1.48(1.13)	1.61(1.24)
**Communication (government, authorities)**									
**Clear & understandable**	1.75(1.29)	1.44(1.22)	2.19(1.16)	1.72(1.27)	1.78(1.35)	1.61(1.28)	2.15(1.33)	1.57(1.24)	1.60(1.30)
**Credible & honest**	1.64(1.30)	1.34(1.18)	2.13(1.18)	1.47(1.28)	1.55(1.31)	1.62(1.33)	2.10(1.37)	1.45(1.27)	1.49(1.26)
**Guided by interests of people**	1.57(1.25)	1.55(1.22)	1.81(1.14)	1.46(1.25)	1.50(1.34)	1.63(1.27)	1.52(1.18)	1.63(1.23)	1.47(1.33)
**Feeling of being … by government, authorities**									
**…well supported…**	1.62(1.23)	1.51(1.15)	1.97(1.16)	1.42(1.22)	1.39(1.27)	1.63(1.24)	1.59(1.17)	1.76(1.17)	1.65(1.32)
**…well informed…**	1.94(1.29)	1.62(1.18)	2.40(1.15)	1.66(1.25)	2.06(1.33)	1.76(1.33)	2.43(1.29)	1.94(1.20)	1.76(1.33)
**…taken seriously…**	1.70(1.25)	1.54(1.19)	2.02(1.19)	1.44(1.28)	1.62(1.24)	1.69(1.27)	1.69(1.18)	1.79(1.23)	1.78(1.33)
**…left alone…**	1.76(1.34)	1.75(1.34)	1.44(1.26)	2.10(1.35)	2.00(1.42)	1.66(1.34)	1.49(1.36)	1.70(1.21)	1.95(1.33)
**Physical health state**	68.46 (20.27)	71.28 (20.07)	66.28 (21.51)	67.55 (20.15)	65.30 (20.34)	71.18 (17.98)	67.61 (21.58)	68.10 (20.07)	70.58 (19.50)
**Positive mental health**	17.38(5.78)	16.90(4.90)	17.91(5.29)	17.58(6.44)	15.77(5.59)	17.52(5.40)	17.38(6.73)	17.27(5.67)	18.85(5.57)

All: N = 7,658; France (FR): N = 940, Germany (GE): N = 917, Poland (PL): N = 924, Russia (RU): N = 986, Spain (ES): N = 960, Sweden (SV): N = 922, the U.K. (UK): N = 1,105, the U.S. (US): N = 904; M = Mean; SD = Standard Deviation.

**Fig 1:**
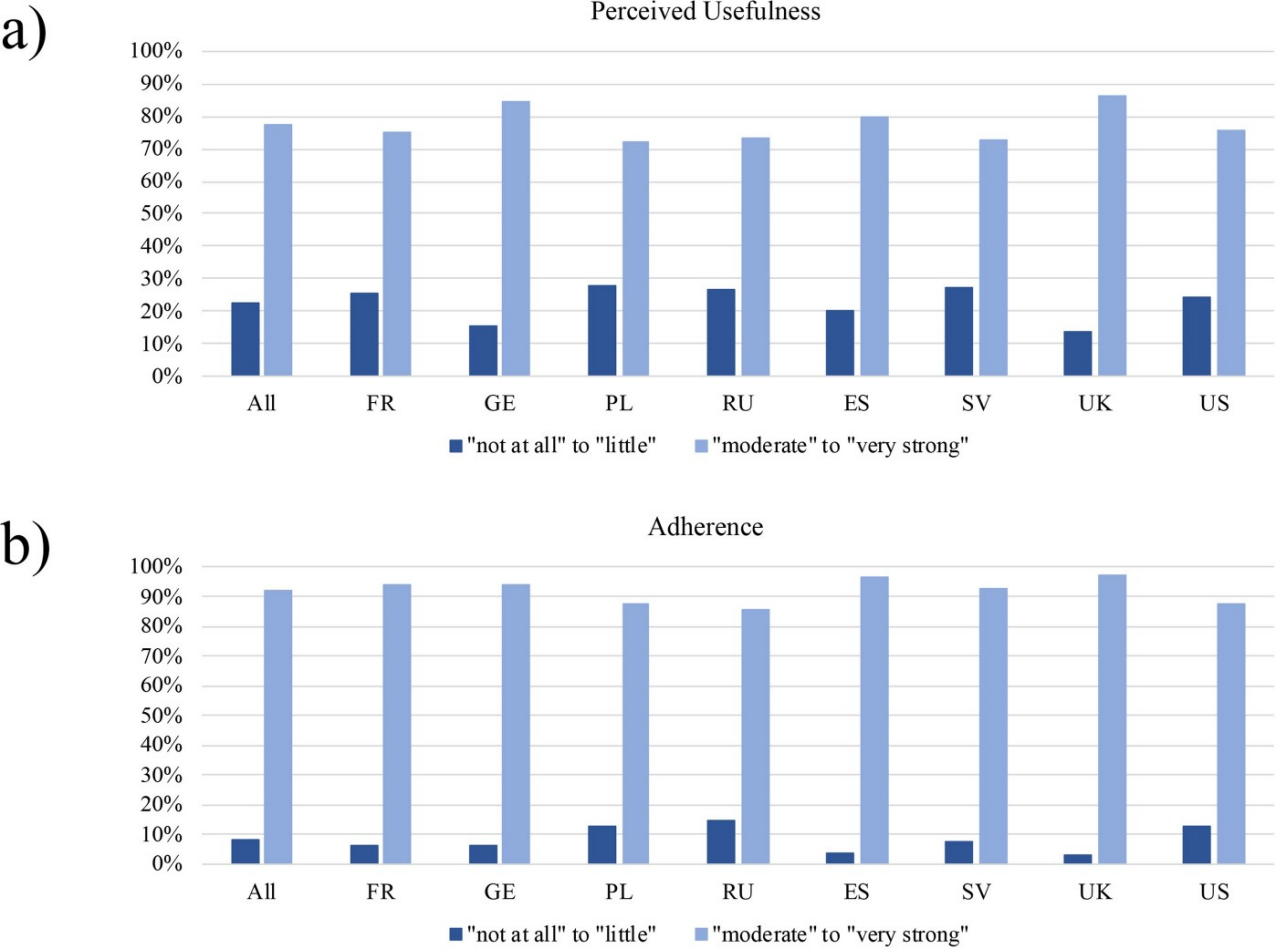
a. Ratings in % of perceived usefulness of measures to combat COVID-19 (total sample and individual country samples); 1b. Ratings in % of adherence to measures to combat COVID-19 (total sample and individual country samples). (All: N = 7,658; France (FR): N = 940, Germany (GE): N = 917, Poland (PL): N = 924, Russia (RU): N = 986, Spain (ES): N = 960, Sweden (SV): N = 922, the U.K. (UK): N = 1,105, the U.S. (US): N = 904).

As shown in Table 3, overall, 5,929 (77.4%) participants rated the measures of the national governments and authorities as useful and 7,026 (91.7%) participants reported adherence to the rules (both: sum of “moderate” to “very strong” ratings). In principle, both variables showed a similar pattern of results in the individual country samples, although gradual differences between countries were also observed. Fig 1A (usefulness) and Fig 1B (adherence) visualize the ratings of both variables (sum of “not at all” to “little” ratings vs. sum of “moderate” to “very strong” ratings).

The subsequent MANOVAs confirmed significant differences between the countries (see Table 5). In the first MANOVA, the Hotelling’s trace was significant, T = .104, F(14,15284) = 56.609, p < .001, η^2^_p_ = .049. The ratings of usefulness and adherence showed significant differences between countries with effect sizes of η^2^_p_ = .030 and .074 indicating a small and a medium effect, respectively. Table 5 provides an overview of the significant results of the pairwise comparisons. The highest mean usefulness rating was found in Germany (significant differences: GE > FR, PL, RU, SV, US), the lowest usefulness rating in France (significant differences: FR < ES, GE, UK, US). The highest adherence was reported in the U.K. (significant differences: UK > FR, GE, PL, RU, SV, US), the lowest in Russia (significant differences: RU < ES, FR, GE, PL, SV, UK, US) (see Tables 3 and 5). The effect sizes (Cohen’s d) of the differences between the countries with the highest and lowest ratings showed a medium effect for usefulness and a strong effect for adherence.

**Table 5:** Pairwise comparisons of the investigated variables between the countries.

	Significant differences (MANOVAs)	Highest Cohen’s d
**Perceived usefulness: F(7,7644) = 33.892, p < .001, η**^**2**^_**p**_ **= .030**	GE > FR, PL, RU, SV, US; ES > FR, PL, RU, SV; UK > FR, PL, RU, SV, US; US > FR, PL, RU, SV	GE > FR: .464
**Adherence: F(7,7644) = 87.727, p < .001, η**^**2**^_**p**_ **= .074**	FR > PL, RU, SV, US; GE > PL, RU, SV, US; PL > RU; ES > FR, GE, PL, RU, SV, US; SV > RU; UK > FR, GE, PL, RU, SV, US; US > PL, RU, SV	UK > RU: .943
**Risk group: F(7,7644) = 22.969, p < .001, η**^**2**^_**p**_ **= .021**	GE > FR, ES, UK; PL > FR, ES, SV, UK; RU > PL, ES, SV, UK; SV > FR; UK > FR; US > ES, SV, UK	US > UK: .354
**Affected: Health: F(7,7644) = 61.772, p < .001, η**^**2**^_**p**_ **= .054**	FR > GE, UK, US; PL > GE, SV, UK, US; RU > FR, GE, PL, ES, SV, UK, US; ES > GE, SV, UK, US; SV > GE, UK, US; US > GE, UK	RU > GE: .814
**Affected: Economically: F(7,7644) = 116.734, p < .001, η**^**2**^_**p**_ **= .097**	FR > D, SV, UK; PL > FR, GE, ES, SV, UK, US; RU > FR, GE, PL, ES, SV, UK, US; ES > GE, SV, UK; US > GE, SV, UK	RU > SV: 1.137
**Affected: Mentally: F(7,7644) = 15.817, p < .001, η**^**2**^_**p**_ **= .014**	PL > FR, GE, RU, UK; RU > GE; ES > FR, GE; SV > FR, GE; UK > GE; US > FR, GE, RU	PL > GE: .464
**Physical health state: F(7,7644) = 14.316, p < .001, η**^**2**^_**p**_ **= .013**	FR > GE, PL, RU, SV; PL > ES; ES > GE, RU, SV, UK; UK > RU; US > GE, PL, RU	FR > RU: .296
**Positive mental health: F(7,7644) = 17.776, p < .001, η**^**2**^_**p**_ **= .016**	GE > FR, RU; FR > RU; PL > FR, RU; ES > FR, RU; SV > RU; UK > FR, RU; US > FR, PL, RU, ES, SV, UK	US > RU: .552
**Communication: Clear & understandable: F(7,7644) = 42.389, p < .001, η**^**2**^_**p**_ **= .037**	GE > FR, PL, RU, ES, UK, US; PL > FR; RU > FR, UK, US; ES > FR; SV > FR, PL, RU, ES, UK, US	GE > FR: .630
**Communication: Credible & honest: F(7,7644) = 50.155, p < .001, η**^**2**^_**p**_ **= .044**	GE > FR, PL, RU, ES, UK, US; RU > FR; ES > FR; SV > FR, PL, RU, ES, UK, US	GE > FR: .669
**Communication: Guided by interests of people: F(7,7644) = 9.063, p < .001, η**^**2**^_**p**_ **= .008**	GE > FR, PL, RU, SV, US; ES > PL; UK > PL; US > ES, UK	GE > PL: .293
**Feel: …well supported: F(7,7644) = 23.133, p < .001, η**^**2**^_**p**_ **= .021**	GE > FR, PL, RU, ES, SV, UK, US; ES > PL, RU; UK > FR, PL, RU, SV; US > PL, RU	GE > RU: .477
**Feel: …well informed: F(7,7644) = 52.837, p < .001, η**^**2**^_**p**_ **= .046**	GE > FR, PL, RU, ES, UK, US; RU > FR, PL, ES, US; SV > FR, PL, RU, ES, UK, US; UK > FR, PL, ES, US	SV > FR: .655
**Feel: …taken seriously: F(7,7644) = 20.138, p < .001, η**^**2**^_**p**_ **= .018**	GE > FR, PL, RU, ES, SV, UK, US; ES > FR, PL; RU > PL; SV > PL; UK > FR, PL, RU; US > FR, PL	GE > PL: .469
**Feel: …left alone: F(7,7644) = 26.281, p < .001, η**^**2**^_**p**_ **= .024**	FR > GE, SV; PL > FR, GE, ES, SV, UK; RU > GE, SV, UK; UK > GE; US > FR, GE, ES, SV, UK	PL > GE: .505

France (FR): N = 940, Germany (GE): N = 917, Poland (PL): N = 924, Russia (RU): N = 986, Spain (ES): N = 960, Sweden (SV): N = 922, the U.K. (UK): N = 1,105, the U.S. (US): N = 904; MANOVA = Multivariate Analysis of Variance; η^2^_p_ = effect size measure of the main effects; Cohen’s d = effect size measure of post-hoc comparisons between countries.

Hotelling’s trace was also significant in the second MANOVA, T = .231, F(42,45824) = 41.943, p < .001, η^2^_p_ = .037. All six variables included in this MANOVA differed significantly between the countries. For being affected economically, there was a medium effect (η^2^_p_ = .097), while the other variables showed small effect sizes (η^2^_p_ = .013 to .054). Tables 4 and 5 show the descriptive statistics and the results of the pairwise comparisons. The effect sizes (Cohen’s d) of the differences between the countries with the highest and lowest ratings showed large effects for being economically and physically affected, a medium effect for positive mental health and small effects for belonging to a risk group, being mentally affected and physical health. For four variables, the worst values were found in Russia (being mentally and economically affected, physical health state, positive mental health), Poland showed the worst result for being mentally affected and the U.S. for belonging to a risk group. Germany showed the best results for being affected in terms of physical and mental health. The U.K. (belonging to a risk group), Sweden (being economically affected), France (physical health state) and the U.S. (positive mental health) each had the best values for one variable.

The third and last MANOVA again showed significant differences (Hotelling’s trace, T = .200, F(49,53454) = 31.134, p < .001, η^2^_p_ = .028) with all seven variables of this analysis differing between the countries (all effect sizes small with η^2^_p_ = .008 to .046). Tables 4 and 5 give the descriptive statistics and the results of the pairwise comparisons. For almost all variables, the most positive responses were found in Germany, while Sweden showed the most positive response for being well informed. Poland and France showed the most negative responses for three variables (PL: communication as guided by interests of people, being taken seriously, being left alone; FR: communication clear and understandable, credible and honest, being well informed) and Russia for one (being well supported). The effect sizes (Cohen’s d) of the differences between the countries with the highest and lowest ratings showed medium effects for 4 variables (communication clear and understandable, credible and honest, being well informed, being left alone) and small effects for 3 variables (communication as guided by interests of people, being well supported and taken seriously).

A shown in Table 3, the correlation between the evaluation of the measures to combat COVID-19 as useful and the adherence to rules was r = .490, p < .001, in the overall sample. Country-specific analyses revealed a range of this relationship between r = .235 (SV) and r = .636 (US), both: p < .001.

Table 6 shows the results of the two hierarchical regression analyses calculated in the overall sample. With regard to the usefulness of the measures (regression 1), more potential predictors became significant and the proportion of explained variance by the predictors was more than twice as high as with regard to adherence (regression 2): 16 vs. 10 predictors and 18.7% vs. 9.2% of the variance. Concerning the usefulness of measures (regression 1), female gender, higher age, having children and a higher social status were significant predictors in Step 1. In Step 2, significant positive predictors were belonging to the risk group, being affected by the COVID-19 situation in terms of physical and mental health, PMH, perception of the governmental communication as clear and understandable, credible and honest, guided by interests of people, the feeling of being well supported and well informed. The variables being affected economically, physical health state and the feeling of being left alone were identified as significant negative predictors (see Table 6).

**Table 6:** Hierarchical regression analyses for perceived usefulness and adherence (total).

	Perceived usefulness				Adherence			
	ß	95% CI	Adjusted R^2^	Changes in R^2^	ß	95% CI	Adjusted R^2^	Changes in R^2^
**Model 1: Step 1, F(6,7651) = 9.944, p < .001, Model 2: Step 1, F(6,7651) = 27.143, p < .001**			.007				.020	
**Gender**	-.045**	[-.155, -.051]			-.105**	[-.254, -.165]		
**Age group**	.071**	[.039, .083]			.101**	[.055, .093]		
**Marital status**	.000	[-.033, .033]			-.024	[-.053, .004]		
**Children**	.031*	[.008, .140]			.013	[-.029, .084]		
**Social status**	.044**	[.023, .071]			.015	[-.006, .035]		
**Living environment**	-.010	[-.048, .019]			.042**	[.025, .082]		
**Model 1: Step 2, F(13,7638) = 131.194, p < .001, Model 2: Step 2, F(13,7638) = 47.502, p < .001**			.187	.181			.092	.073
**Risk group**	.048**	[.059, .169]			.040**	[.031, .131]		
**Affected: Health**	.065**	[.041, .088]			.071**	[.040, .082]		
**Affected: Economically**	-.027*	[-.045, -.003]			-.025^(^*^)^	[-.039, .000]		
**Affected: Mentally**	.144**	[.119, .167]			.230**	[.174, .218]		
**Communication: Clear & understandable**	.046*	[.008, .074]			-.023	[-.048, .013]		
**Communication: Credible & honest**	.133**	[.082, .154]			-.007	[-.038, .027]		
**Communication: Guided by interests of people**	.072**	[.037, .096]			.062**	[.022, .076]		
**Feel: …well supported**	.043*	[.008, .073]			.019	[-.014, .045]		
**Feel: …well informed**	.089**	[.049, .111]			.048*	[.009, .065]		
**Feel: …taken seriously**	.026	[-.008, .057]			.016	[-.017, .042]		
**Feel: …left alone**	-.087**	[-.095, -.054]			-.047	[-.053, -.016]		
**Physical health state**	-.025*	[-.003, .000]			-.010	[-.002, .001]		
**Positive mental health**	.039**	[.003, .013]			.080**	[.009, .018]		

All: N = 7,568; gender: 0 = woman, 1 = man; ß = standardized coefficient beta; CI = Confidence Interval;

**p < .01,

*p < .05,

^(^*^)^p < .06.

Looking at adherence to the measures (regression 2), female gender, higher age and living environment (living in a rural community) were positive predictors in Step 1. In Step 2, belonging to the risk group, being affected by the COVID-19 situation in terms of physical and mental health, PMH, perception of the governmental communication as guided by interests of people and the feeling of being well informed served as positive predictors. Being affected economically served as a (significant) negative predictor. In both models, being affected in terms of mental health was the strongest predictor (see Table 6).

Table 7 provides a simplified overview of the two hierarchical regression analyses calculated separately for each country-specific sample. For both outcomes, each sample showed a specific pattern of results that differed from the other samples. Although the number of significant predictors for usefulness of measures (regression 1) was remarkably higher in the overall sample than for adherence to rules (regression 2), this finding (i.e., 16:10) was approximately replicated only in Germany (12:8). In all other countries, however, the number of significant predictors for the two variables was roughly balanced. In contrast, similar to the overall sample, the proportion of the variance explained by the predictors was substantially higher for usefulness of measures than for adherence in all countries except the US. It varied remarkably between the investigated countries (see Table 7). In terms of usefulness (regression 1), the proportion was highest in Sweden with 42.3%, which was almost four times higher than in the U.K. (10.8%). For adherence (regression 2), the highest proportion was in Russia (19.9%) and the lowest in Spain (7.5%); in the U.K., the model for adherence was not significant. Overall, the findings show that mental (6 times: FR, GE, PL, SV, UK, US) and physical health (5 times: FR, GE, PL, RU, US) as well as the feeling of being well informed (5 times: FR, GE, PL, RU, UK) were the most common significant predictors of the perceived usefulness of measures to control COVID-19. The most frequent significant predictors of adherence were female gender (8 times: all countries), being affected in terms of mental health (8 times: all countries), age (7 times: ES, FR, GE, PL, SV, UK, US), and the feeling of being well informed (6 times: ES, FR, GE, PL, RU, SV).

**Table 7:** Simplified presentation of separate hierarchical regression analyses for perceived usefulness and adherence for the eight countries.

	Measures usefulness								Adherence to rules							
	FR	GE	PL	RU	ES	SV	UK	US	FR	GE	PL	RU	ES	SV	UK	US
**Step 1**																
**Gender**					-X	-X			-X	-X	-X	-X	-X	-X	-X	-X
**Age group**		X	X		X		X		X	X	X		X	X	X	X
**Marital status**		-X				X							-X	X		
**Children**				(-X)							-X					
**Social status**	X	X		X			X		X			X				
**Living environment**								-X								-X
**Step 2**																
**Risk group**	X	X		X					X	X		(X)				
**Affected: Health**	X	X	X	X				X				X		X	X	X
**Affected: Economically**		-X	X						X		X	X				
**Affected: Mentally**	X	X	X			X	X	X	X	X	X	X	X	X	X	X
**Communication: Clear & understandable**			X	X								X				
**Communication: Credible & honest**	X	X			X	X				X						
**Communication: Guided by interests of people**				X	X	(X)	X									
**Feel: …well supported**					X		X							-X		
**Feel: …well informed**	X	X	X	X			X		X	X	(X)	X	X	X		
**Feel: …taken seriously**		X				(X)		-X						X		
**Feel: …left alone**	-X	-X			-X	-X				-X			-X			
**Physical health state**		-X								-X						
**Positive mental health**								X			X		X	X		
**Overall adjusted R**^**2**^ **(%)**	**24.3**	**37.8**	**23.1**	**29.7**	**26.8**	**42.3**	**10.8**	**12.1**	**8.6**	**19.5**	**19.4**	**19.9**	**7.5**	**14.3**	**n.s.**	**13.9**

France (FR): N = 940, Germany (GE): N = 917, Poland (PL): N = 924, Russia (RU): N = 986, Spain (ES): N = 960, Sweden (SV): N = 922, the U.K. (UK): N = 1,105, the U.S. (US): N = 904; gender: 0 = woman, 1 = man; -X = significant negative predictor, X = significant positive predictor, both:**p < .01 and *p < .05; (-X)/(X) = ^(^*^)^p < .06.

The regression analyses revealed the following country-specific results for predicting perceived usefulness: The strongest predictors were being affected in terms of mental health in France (FR: ß = .168, 95% CI [.096, .229], p < .001) and the US (ß = .250, 95% CI [.173, .329], p < .001), older age in Germany (ß = .283, 95% CI [.186, .316], p < .001) and Poland (ß = .177, 95% CI [.081, .204], p < .001), being affected in terms of physical health in Russia (ß = .285, 95% CI [.212, .338], p < .001), the perception of governmental communication as credible and honest in Spain (ß = .202, 95% CI [.076, .272], p < .01) and Sweden (ß = .284, 95% CI [.157, .341], p < .001) and the feeling of being well supported in the U.K. (ß = .151, 95% CI [.049, .209], p < .01).

The following country-specific results were found for predicting adherence: In France, Poland, Spain, Sweden and the U.S. being affected in terms of mental health was the strongest predictor (FR: ß = .191, 95% CI [.096, .222], p < .001; PL: ß = .293, 95% CI [.198, .340], p < .001; ES: ß = .155, 95% CI [.061, .173], p < .001; SV: ß = .253, 95% CI [.144, .268], p < .001; US: ß = .271, 95% CI [.179, .321], p < .001). In Germany, age was the strongest predictor (ß = .283, 95% CI [.130, .234], p < .001) and in Russia, being affected in terms of physical health (ß = .244, 95% CI [.143, .257], p < .001). In the U.K., the strongest predictor was female gender (ß = -.100, 95% CI [-.256, -.066], p < .01), but the model was not significant.

## Discussion

The COVID-19 pandemic has significantly changed the daily lives of many people around the globe in recent months. Many governments have introduced extraordinary measures ranging from recommendations to avoid public places and travel to entry bans and “stay-at-home orders”. Wearing facemasks and maintaining social distance became part of everyday life in many countries [3]. In view of the lack of specific drug therapies or vaccinations, behavioral measures are the key countermeasures in the fight against COVID-19. Their success depends largely on their evaluation by the population as useful and the willingness to adhere to them.

The present study provides one of the first representative results on the evaluation of the usefulness of and adherence to early introduced anti-COVID-19 measures and their predictors from eight different countries. Overall, as well as in the eight individual national samples, about three-quarters of the participants considered the government measures introduced to be useful (Research Question 1) and more than 90% of the participants (exceptions RU 85%, US 87%, PL 88%) reported adherence to the measures (Research Question 2). Looking at the worldwide COVID-19 statistics [2], the spread of the virus and the daily case numbers did not increase significantly in the months of June and July 2020 in most of the countries studied–with the notable exception of the U.S. Rather, there was a decrease or slight stagnation on a country-specific plateau that was remarkably lower than the peak reached in most countries between March and May of 2020. It can be assumed that the high level of adherence to the regulatory measures identified in the present study has contributed significantly to slowing down the spread of COVID-19. The predominantly positive assessment of the measures could also have contributed to this success. However, the present results underline that perceived usefulness and adherence should not be equated. Although both were positively correlated, their correlation was only r = .49 and also varied considerably between countries (see Table 3). Most people were willing to adhere to the rules, even if not all of them considered the measures to be useful. This discrepancy can be explained, at least in part, by the theory of planned behavior [55], which describes the attitude towards an action as an important–but not the only–antecedent of individual behavior. According to this theory, behavioral intention and perceived behavioral control are the most important decisive factors that predict behavior. Therefore, it is urgent that governmental communication enhances the sense of control among the population. It should explain the sense of the anti-COVID-19 measures (including health precautions [18]) and emphasize that by adhering to them everyone can effectively contribute to the fight with the pandemic and support the healthcare system. It is important that the introduced measures are not perceived as an annoying obligation enforced by the authorities.

Comparing countries revealed significant differences for all 15 variables in the three MANOVAs conducted (see Tables 3–5). Germany showed the most positive picture: highest rating for the usefulness of the measures; perception of government COVID-19 communication as clear and understandable, credible and honest, guided by people's interests; feeling of being well supported and taken seriously; lowest values for impairment in terms of physical and mental health and feeling left alone. During the springtime, Germany has been relatively successful in dealing with the pandemic, which could have led to the generally more positive assessment of the COVID-19 situation in Germany compared to the other countries studied. The population subjectively felt less affected by COVID-19 and was more satisfied with the government's measures. As revealed by the present findings, the Russian population felt more affected by the COVID-19 situation than the populations of the other countries: highest ratings for the impact in terms of physical health and in economic terms. In France and in Poland the population was less satisfied with the state COVID-19 communication (FR: lowest level of communication perception as clear and understandable and as credible and honest; lowest feeling of being well informed; PL: lowest level of communication perception as being guided by people's interests; lowest feeling of being well supported and taken seriously; highest feeling of being left alone). These results can provide the authorities in the countries concerned with targets for improvement. They can for example explain more explicitly the sense of the introduced measures in public communication. Additionally, they are recommended to conduct representative surveys to assess the most important needs and concern of the population to improve the next governmental anti-COVID-19 steps. This can reduce the feeling of being left alone during the pandemic. Overall, it can enhance the trust in and the satisfaction with the governmental actions which both are important predictors of adherence to the measures [41,56].

Further information in this respect is provided by the results of the regression analyses. Overall, the regressions showed a better prediction for the evaluation of usefulness than for adherence to the measures across all countries and in the individual countries with the exception of the USA. In general, the results of the regressions are in line with the PMT [19], according to which a stronger assessment of threat and effectiveness and a lower cost perception should be positive predictors for the evaluation of benefits and adaptation to recommended behavior [22,24,25]. In our overall sample (see Table 6), belonging to the COVID-19 risk group, lower perceived physical health and a higher level of experienced physical and mental consequences of the COVID-19 outbreak were significant predictors of both outcomes, confirming Hypothesis 1. All these variables promote the perception of one’s own vulnerability and should therefore contribute to the assessment of the threat. Furthermore, in line with previous findings [34,57], it was confirmed that better PMH predicts higher response- and self-efficacy, further confirming Hypothesis 1. Individuals with better PMH often consider extraordinary situations as interesting challenges with less perceived costs and find unusual ways to cope with them [58]. Thus, it can be assumed that the enhancement of the individual PMH level can contribute to higher adherence to the measures. In a recent study, physical activity (e.g., jogging and cycling) has been reported to foster the level of PMH [59]. This finding is in line with available literature [60] and the recommendation of the World Health Organization to engage at least for 30 minutes a day in moderate physical activity to improve mental and physical health [61]. Thus, governmental communication and future anti-COVID-19 steps are recommended to focus on the increase of physical activity among the population. It should be emphasized that each individual–except people with serious physical disabilities–can engage in daily physical activity for at least 30 minutes in the own rhythm according to the individual physical conditions outdoors and at home [62]. Examples of physical activities and specific exercises that do not require expensive equipment can be provided in advertising campaigns on TV, officials governmental online sites and billboards. This can be beneficial for all investigated countries, but specifically for Russia where our present findings revealed the lowest level of PMH and of adherence to the anti-COVID-19 measures. The increase of both variables can reduce the burden on the healthcare services which are overloaded in times of COVID-19.

Other earlier findings have shown that well-organized public communication can promote the perceived effectiveness of measures in the population and reduce their perceived costs [20]. Consistent with this, the perception of government communication as guided by the interests of people and the feeling of being well informed proved to be further positive predictors of perceived usefulness and adherence. In addition, perceived usefulness was also positively predicted by the perception of government communication as clear, understandable, credible and honest and by the feeling of being well supported, partially confirming Hypothesis 1. A negative predictor of usefulness was the feeling of being left alone, confirming Hypothesis 2 only for usefulness. Interestingly, the feeling of being taken seriously was unrelated to both perceived usefulness and adherence, contradicting Hypothesis 1. Being affected economically by the COVID-19 situation, however, negatively predicted both outcomes, confirming Hypothesis 2. This could be explained by the fact that economic disruptions are immediately noticeable, while COVID-19 is less tangible in the future. It is therefore important for governments to address economic problems in an immediate manner not only for social reasons, but also to increase the likelihood of implementing the behavioral measures. In many countries, the governmental measures included the temporary closing of non-essential businesses and bans on cultural events. Specifically, self-employed people including artists (actors, vocalists) were negatively affected by this action. In accordance with present findings, these measures led to reactance and public protests in some countries such as the U.S. [63]. To support the affected people and small companies, many governments and authorities provided financial grants and subventions, for example Germany and Russia [64,65]. In the U.S., first non-essential businesses re-opened already in April [63].

Even though demographic variables were not the focus of the present study, it must be mentioned that both outcomes were positively predicted by female gender and higher age. This is in line with previous studies on unhealthy and risky behavior [66,67] and underlines that younger males in particular are at increased risk of refusing to adhere to government measures. Higher levels of the personality trait sensation-seeking and lower risk perception could explain this finding [68]. Therefore, this group should be given special consideration in further government communication and in programs to combat the spread of COVID-19. For example, governmental communication in all countries can explicitly emphasize that even though younger males may believe to be at less risk to come down with COVID-19, they can be carrier of the virus and unconsciously infect their grandparents and other older people who belong to the high-risk group. Therefore, they should be pointed to their enhanced responsibility for the reduction but also the spread of the pandemic by advertising campaigns on different media.

Following the regression analyses calculated with the total sample, the eight country-specific samples were considered separately (see Table 7). These analyses showed partly consistent, but also partly country-specific patterns of results that deviated from the results of the overall sample and in some cases indicated contradictory relationships between variables in different countries. It is remarkable that overall the variables being affected in terms of mental health–a factor that can enhance threat appraisal, vulnerability–and the feeling of being well informed–a factor that can enhance efficacy–belonged to the most frequent predictors of both outcomes across countries [22]. These results that are in line with other recently published findings from cross-national research [56] emphasize the urgent importance of competent information by governments and authorities. They also underline the need for further investments in mental health services, including low-threshold programs–not only to support those already affected and to prevent a future increase in mental disorders, but especially with regard to COVID-19, to improve adherence to behavioral interventions to control the pandemic. All countries, but especially Poland where the variable being affected in terms of mental health was the highest and Russia where the lowest adherence level was found, could profit from further investments in low-threshold mental health services and intervention programs to reduce the risk of mental problems. In the longer-term, this investment can ease the burden of the healthcare system. This conclusion is confirmed by the following results.

In terms of differential findings, age proved to be the strongest predictor of both outcomes in Germany and of perceived usefulness in Poland. In both countries, being affected mentally was also a strong predictor of adherence and perceived usefulness. However, the Polish participants showed particularly negative and the German participants very positive values for this predictor and both outcomes. In addition, the Polish participants had below-average scores for a number of variables (being affected physically, economically and mentally, governmental communication clear and understandable, being well-in), which were predictors of both outcomes and therefore could be potential targets for interventions to improve the active participation of the population in the measures against COVID-19 in this country. The present findings emphasize the need of further investments in low-threshold mental health services and a more comprehensive governmental communication especially in Poland. In contrast, the German sample showed the most positive responses compared to the other countries for a number of factors which in this country were significant predictors for either both outcomes (being affected mentally, governmental communication credible and honest, feeling of being left alone) or just for perceived usefulness (being affected in terms of physical health, feeling of being taken seriously). This may at least partly explain the high level of perceived usefulness in Germany. On the other hand, Germany did not have the highest level of adherence which means that other factors are important and should be identified in order to further enhance adherence in this country. Moreover, to foster the translation of usefulness perception to adherence, the sense of individual control of the current situation should be specifically emphasized in the governmental communication in Germany.

In Russia, being affected physically and in France, being affected mentally were the strongest predictors of both outcomes. However, the Russian participants had the most negative ratings for being physically affected, while the French had the second highest positive rating for being mentally affected. Because both of these variables may be more difficult to change, it is interesting to note, that there were two other variables where the French sample had the lowest scores compared to other countries (feeling of being well informed and the perception of the governmental communication as credible and honest) and that also significantly predicted perceived usefulness. Improving these variables could therefore represent viable targets for improving population participation in the anti-COVID-19 measures in France where perceived usefulness was the lowest of all countries. In contrast, the Russian sample showed the lowest adherence ratings, and potentially modifiable predictors for these were government communication (clear and understandable) and the feeling of being well informed. Thus, both countries can profit from a more comprehensive governmental communication that explains the importance of the collective adherence to the measures and that emphasizes the positive effect of physical activity for both physical and mental health [62]. To stress the sense of the introduced measures, the authorities are recommended to present potential prospective COVID-19 infection and death statistics in case of a collective adherence vs. a collective not-adherence to the measures. This could increase the feeling of being well informed and foster the collective threat appraisal among the population that both are important for adherence to the measures.

In Spain, the U.S. and Sweden, the strongest predictor of adherence was being affected mentally (Spain and U.S.: highest and second highest values, Sweden: average). In all three countries, younger people showed lower adherence. Thus, the governmental communication and anti-COVID-19 programs of the three countries should specifically focus on this population group and stress its responsibility for the success of the pandemic fight. Moreover, the feeling of being well informed was a predictor in Spain and Sweden. In this variable the Swedish participants showed the highest values of all countries while the Spanish sample scored slightly below average. Interestingly, Spain showed the second highest rating for adherence, whereas Sweden and the U.S. were average in this respect. This can perhaps be at least partly explained by the higher relative death toll experienced in Spain at the time of data collection for the present study. Perceived usefulness was most strongly predicted by the assessment of governmental communication as credible and honest in Sweden (second highest value) and Spain (average), but not in the U.S. Even though, by far, most COVID-19 cases were registered in the U.S., participants from this country did not show the highest subjective levels of being affected physically, mentally and economically. To the contrary, the U.S. sample did report the highest level of PMH. This variable is known to confer resilience and to reduce the probability of negative outcomes such as mental disorders and suicide [29–31]. Therefore, it can be assumed that the high PMH level also serves as a protective factor in the U.S. population in the current COVID-19 situation. This finding emphasizes the potential benefits of a cross-national enhancement of the PMH level. Against this background, all authorities are recommended to stress the importance of physical activity–that can foster PMH [59]–in public communication and by using advertising campaigns.

Since Sweden and the U.S. had only average or slightly below average levels of adherence and perceived benefits, the question remains as to how these could be improved. Given that the U.S. scored below average on all three variables related to government communication (but Sweden only for one: guided be the interests of people), one might think that this could be a starting point for improvements. However, none of these variables were significant predictors in the U.S. Focusing on changing the attitudes and behaviors of young people might be an alternative, albeit a difficult one. Especially advertising campaigns on social media such as Facebook and Twitter that are regularly used by younger people [69] could be an effective strategy to reach this population group. Sweden is another special case in that the population does not seem to feel particularly affected in terms of health and is mostly very positive about its government’s communication, but the excess mortality rate was one of the highest among the countries at the time of data collection in our study. This could be explained by the observation that the Swedish participants gave the highest rating for being economically affected. This may represent a higher cost perception, which according to PMT [19] may have a negative impact on perceived benefits and willingness to adapt. Therefore, a stronger economic support by the government is recommended especially in Sweden.

Finally, in the U.K., there was no significant prediction of adherence and only a very low prediction for perceived usefulness. Given that the U.K. participants showed the highest ratings for adherence and the second highest for usefulness, this could be explained by a ceiling effect. Looking at the absolute levels of the mean ratings, this explanation seems more convincing for adherence (mean of 3.35 on a 0–4 scale) than for usefulness (mean of 2.51 on the same scale). The high level of adherence might also be related to the exceptionally high morbidity rate of the U.K. and the fact that the country’s prime minister was severely affected by COVID-19, both of which should lead to higher threat appraisal according to the PMT. The small prediction of usefulness was most strongly driven by the feeling of being well supported (second highest rating after Germany) and also by feeling well informed (average rating) and perceiving government communication as guided by people’s interests (together with Spain second highest rating behind Germany). Future studies are suggested to investigate further potential predictors of adherence to the measures specifically in the U.K.

Despite the timeliness of the current study, the following limitations should be taken into account. First, due to the very dynamic circumstances, the present results represent a snapshot of the COVID-19 situation in the summer of 2020 in the eight countries studied. Secondly, due to the cross-sectional design of the current study, only hypothetical causality considerations are possible. Third, age, gender and region stratification were implemented to achieve representativeness of the data used in the present study. To increase the representativeness, future research is suggested to include further variables for the stratification such as marital status, level of education and income. Fourthly, in order not to overload the participants, only a limited number of potential predictors of perceived usefulness and self-reported adherence were examined. In order to obtain a more comprehensive picture of the current COVID-19 situation, future studies should include actual behavior measures of adherence and additional factors that may influence attitudes and behavior, e.g. personality traits such as sensationalism [70], self-esteem [71], fear [19], seas of control [16], and past experience with extraordinary situations [72]. Moreover, future studies are recommended to use a recently developed ten-item instrument COVID-SCORE-10 that assesses the individual evaluation of the governmental communication and actions towards COVID-19 [56]. This instrument that was published after our data collection can contribute to further prediction of the population adherence to the anti-COVID-19 measures and thus complement our present findings. Fifthly, no Asian (despite part of Russia), African or South American countries were included in the present study, which limits the generalizability of the current findings. Given the differences in the timeline and the extent of COVID-19 dissemination and governmental action [2,3], future studies will have to consider whether the current findings can be generalized to countries from these regions of the world.

## Conclusions

To conclude, the present cross-national study shows that across various countries, government measures to combat COVID-19 are mostly considered useful and that there is a very high rate of adherence. The lowest adherence was reported in Russia and Poland, where people felt particularly left alone and not well supported, and from the U.S. and Sweden, where the governments had at least an ambivalent attitude towards behavioral anti-COVID-19 measures. The highest levels of adherence were reported in countries that either had very high mortality rates (U.K., Spain, France) or where government communication was considered particularly positive (Germany). In addition, the perceived usefulness of and adherence to anti-COVID-19 measures are predicted by various factors, such as female gender, higher age, being mentally affected and the quality of government communication with respect to COVID-19. Some of these relationships are universal, others more country-specific. Therefore, each government must address the specific pattern for its population in order to successfully fight the pandemic. Moreover, the governmental communication in all countries should enhance the sense of control among the population. It should emphasize that the adherence to the anti-COVID-19 measures by each individual is important for the fight with the pandemic. It is hoped that further investigation of the predictors and their (country-specific) modification will contribute to the successful management of the COVID-19 pandemic. Especially longitudinal research that focuses on potential predictors of adherence is of great importance considering the permanently changing COVID-19 situation around the globe.

## Supporting information

S1 Abbreviations List of abbreviations. (DOCX)

S1 Dataset Dataset used for analyses in present study. (SAV)
