# Supplementary material for: Behavioral measures to fight COVID-19: An 8-country study of perceived usefulness, adherence and their predictors
Source: PLoS One. 2020 Dec 7;15(12):e0243523. doi: 10.1371/journal.pone.0243523 (PMC7721173; doi:10.1371/journal.pone.0243523)
Supplement: S1 Abbreviations — (DOCX) [file pone.0243523.s001.docx]

**List of Abbreviations**

CI – Confidence Interval

COVID-19 – severe acute respiratory syndrome coronavirus 2, SARS-CoV-2

d – Cohen’s d, effect-size measure of the post-hoc comparisons

EQ VAS – EuroQuol Visual Analogue Scale

ES – Spain

FR – France

GE – Germany

M – Mean

MANOVA – Multiple Analyses of Variance

p – Significance

PL – Poland

PMH – Positive Mental Health

PMT – Protection Motivation Theory

r – correlation

RU – Russia

SD – Standard Deviation

SV – Sweden

T – Hotelling’s trace

U.K. – United Kingdom

U.S. – United States

α – Cronbach’s α, reliability measure

ß – standardized coefficient ß

η^2^_p_ – Partial eta squared, effect-size measure of the main effect
